# Implementation of evidence-based alcohol policies to reduce alcohol-related harm and liver disease to advance public health in Europe

**DOI:** 10.1016/j.lanepe.2026.101707

**Published:** 2026-05-25

**Authors:** Elisa Pose, Luis Antonio Díaz, Richard Parker, Mads Israelsen, Thomas Marjot, Ewan Forrest, Ashwin Dhanda, Catherine Paradis, Juan Pablo Arab, Aleksander Krag, Ramón Bataller, Frank Murray, Jeffrey V. Lazarus, Patrizia Carrieri, Paul N. Brennan

**Affiliations:** a Liver Unit, Hospital Clínic of Barcelona, Fundació Clínic per a la Recerca Biomédica (FCRB) - Institut d’Investigacions Biomèdiques August Pi i Sunyer (IDIBAPS), Barcelona, Spain; b Centro de Investigación Biomédica en Red de Enfermedades Hepáticas y Digestivas - CIBERehd, Spain; c MASLD Research Center, Division of Gastroenterology and Hepatology, University of California San Diego, San Diego, CA, USA; d Departamento de Gastroenterología, Escuela de Medicina, Pontificia Universidad Católica de Chile, Santiago, Chile; e Leeds Liver Unit, Leeds Teaching Hospitals NHS Trust, Leeds, United Kingdom; f Centre for Liver Research, Department of Gastroenterology and Hepatology, Odense University Hospital, Odense, Denmark; g Institute for Clinical Research, University of Southern Denmark, Odense, Denmark; h The Roger Williams Institute of Liver Studies, Faculty of Life Sciences & Medicine, King's College London, United Kingdom; i Department of Gastroenterology Glasgow Royal Infirmary, Glasgow, United Kingdom; j University of Glasgow, Glasgow, United Kingdom; k Peninsula Medical School, Faculty of Health, University of Plymouth, Plymouth, United Kingdom; l WHO Regional Office for Europe, Marmorvej 51 2100, Copenhagen, Denmark; m Division of Gastroenterology, Hepatology, and Nutrition, Department of Internal Medicine, Virginia Commonwealth University School of Medicine, Richmond, VA, 23298, USA; n Department of Medicine, Royal College of Surgeons in Ireland, Beaumont Hospital, Dublin 9, Ireland; o City University of New York Graduate School of Public Health and Health Policy (CUNY SPH), New York, NY, USA; p Barcelona Institute for Global Health (ISGlobal), Barcelona, Spain; q Faculty of Medicine and Health Sciences, University of Barcelona, Barcelona, Spain; r Chair, *The Lancet Regional Health – Europe* Series on chronic liver disease in Europe; s Aix Marseille Univ, INSERM, IRD, SESSTIM, Sciences Économiques & Sociales de la Santé & Traitement de L'information Médicale, ISSPAM, Marseille, France; t Division of Molecular and Clinical Medicine, University of Dundee, Dundee, Scotland, United Kingdom

**Keywords:** Ethanol, Alcohol use disorder, Policy, Screening, Industry, Alcohol-related liver disease, Alcohol-associated liver disease, Cirrhosis, Epidemiology, High-risk population

## Abstract

Alcohol-related liver disease (ALD) remains a leading cause of preventable morbidity and mortality in Europe. Despite robust evidence that alcohol-related population-level policies delay use initiation and reduce associated harms, their implementation in Europe has been inconsistent and frequently undermined by alcohol industry interference, fragmented governance, and policy inertia. The burden of ALD as well as combined metabolic dysfunction and alcohol-associated liver disease (MetALD) has grown steadily, driven by increased alcohol intake, widespread metabolic risk factors, delayed diagnosis, and poor integration between primary care, substance use services, endocrinology, and hepatology. In this Series paper, we combine epidemiology, policy evaluations, and clinical evidence to examine these findings through the lenses of policy, system preparedness, education, and stigma. We highlight screening strategies for alcohol use and liver disease and describe models of multidisciplinary and digital care. Finally, we outline priorities for policy reform, stigma reduction, youth-focused prevention, and research on biomarkers, pharmacotherapies, and digital/artificial intelligence tools.

## Introduction

**Fig. 1:**
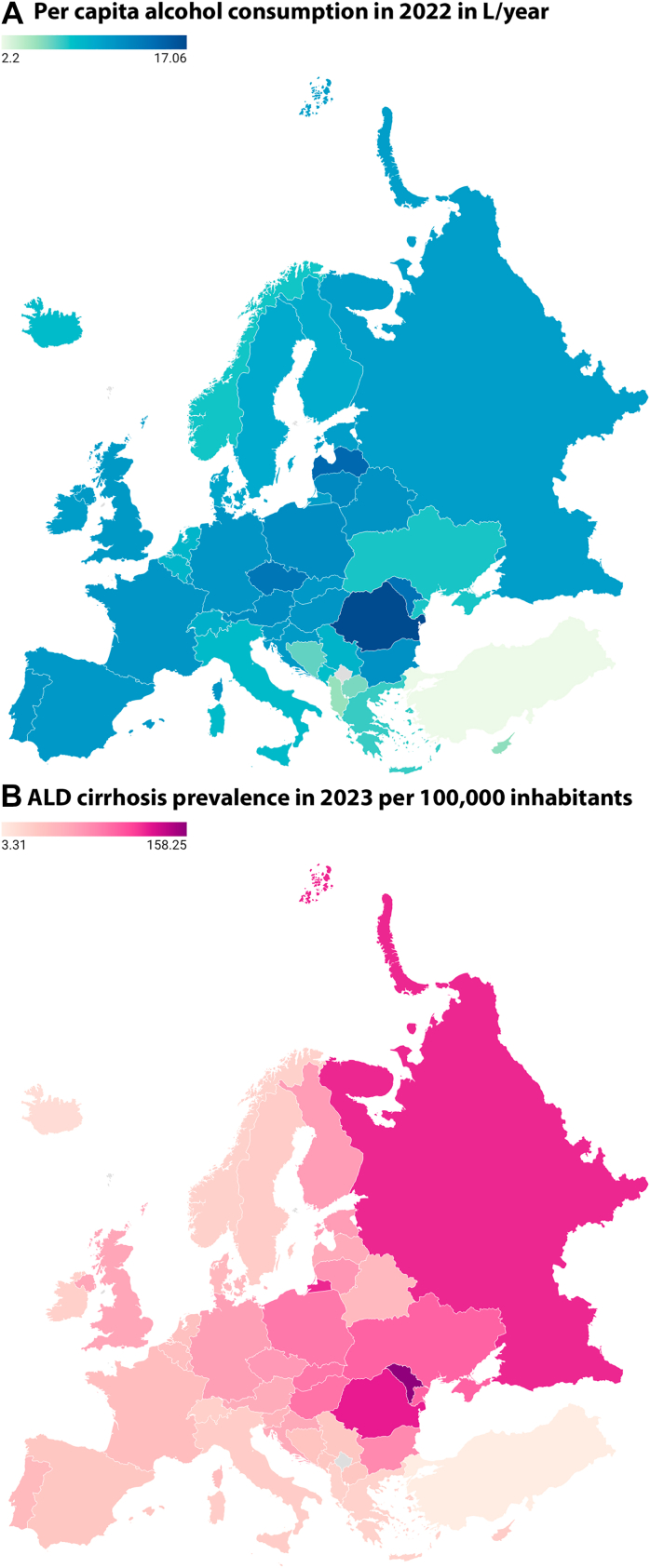
(A) Per capita (of people aged ≥15 years) alcohol consumption (in L of pure alcohol) in Europe in 2022, calculated using the average intake between 2020 and 2022. (B) Age-standardised prevalence of alcohol-related cirrhosis in Europe in 2023 (per 100,000 inhabitants).

The global burden of alcohol-related harm is high, including major health, social, and economic consequences, such as crimes, road traffic injuries, and loss of productivity.^1^ Europe is the World Health Organisation (WHO) region with the highest alcohol consumption and related harms and deaths (Fig. 1A),^1^ with males and females consuming 21.0 and 7.3 L of pure alcohol per capita yearly (equivalent to 45.5 and 15.7 g daily, respectively).^1^ This volume of alcohol intake contributes to more than four years of life lost for men who use alcohol in the EU.^2^ Moreover, alcohol is responsible for roughly one out of every eleven deaths across Europe.^3^

**Key messages**

• Europe faces an enormous preventable burden of ALD and MetALD, driven by excessive alcohol consumption, late diagnosis, industry obfuscation and insufficient policy enforcement despite increasing evidence of effective interventions.
• Implementation gaps are the primary obstacle to reducing alcohol-related harm, with strong evidence showing that taxation, minimum unit pricing, and marketing restrictions remain underutilised or inconsistently applied across Europe.
• In parallel, implementation research to evaluate the long-term impact of the various school-based educational approaches to reduce risky patterns of alcohol use is needed.
• Preparedness within healthcare systems remains limited, with poor integration within and between clinical systems leading to missed opportunities for timely detection, intervention, and treatment of alcohol use disorder and ALD and/or MetALD.
• Stigma, social inequities, and low health literacy hinder help-seeking, delay diagnosis, and compromise care; targeted educational initiatives—linking training, lived-experience engagement, and public awareness—are crucial to overcoming these barriers.
• A coordinated European response, combining evidence-based policy, timely detection, multidisciplinary and digital models of care, and community engagement can kerb the growing burden of ALD and MetALD and improve liver health outcomes.

**Search strategy and selection criteria**

We searched PubMed/MEDLINE to identify potential alcohol-related public health policies and strategies. Search strings were built based on: (1) (“policy” OR “health policies”) AND (2) (“alcohol”). We searched for studies published from inception through October 8, 2025. We also drew on key sources from 2005 onwards, including major journals in hepatology and public health, the World Health Organisation European Region, the Organisation for Economic Co-operation and Development, and the European Commission. Evidence was selected iteratively for relevance to four domains—policy, preparedness, education, and stigma—and to illustrative European case studies (e.g., minimum unit pricing, advertising restrictions). We prioritised high-quality reviews, meta-analyses, large cohort studies, and authoritative policy evaluations. The final version of the manuscript was drafted through multidisciplinary, iterative consensus among hepatology, substance use, and public-health experts. No specific funding was received; the views expressed are those of the authors and not necessarily of their institutions.

In parallel, the global prevalence of alcohol-related liver disease (ALD) and related primary liver cancer has continued to increase over the past three decades (Fig. 1B).^4^ In Europe, ALD now accounts for approximately 10 deaths per 100,000 inhabitants.^5^ Beyond the devastating personal and societal consequences, ALD is placing an increasing strain on healthcare systems.^6^ ALD-related disability-adjusted life years have also risen markedly, contributing to liver disease now being the second leading cause of working years lost in Europe, after ischaemic heart disease.^7^ This increase in ALD is deeply concerning, and has occurred despite recent national and international harm-reduction initiatives.^7^

In 2025, the political declaration of the United Nations General Assembly fourth high-level meeting on non-communicable diseases (NCDs) reiterated the need for an integrated, comprehensive, and people-centred approach to prevention and control, including alcohol-related harms. The magnitude of harm caused by ALD underscores the urgent need for decisive action and sustained implementation.

This Series paper summarises the current landscape of alcohol-related harm and highlights persistent shortcomings in public health initiatives to reduce ALD. Although it focuses on ALD, the public health burden of alcohol extends far beyond the liver, contributing to multiple additional health and social harms.^1^ We also review emerging evidence on screening strategies for alcohol use disorder (AUD) and ALD, along with different models of multidisciplinary care. Finally, we evaluate the alcohol policy preparedness of different European countries and propose new measures and strategic approaches to strengthen prevention and reduce the burden of ALD.

## Current gaps in alcohol-related policies, preparedness, and education

### Current policy landscape of alcohol control in Europe

In 2018, WHO launched the SAFER framework, advocating for five high-impact strategies (i.e., “best buys”) that can help governments to reduce alcohol use and its health, social, and economic consequences, including: (1) strengthening restrictions on alcohol availability; (2) advancing and enforcing drink-driving countermeasures; (3) facilitating access to screening, brief interventions, and treatment; (4) enforcing bans or comprehensive restrictions on alcohol advertising, sponsorship, and promotion; and (5) raising alcohol prices through excise taxes and pricing policies.^8^ Subsequently, WHO launched the Global Alcohol Action Plan 2022–2030, which was endorsed in May 2022 by Member States to reduce the harmful use of alcohol worldwide by implementing high-impact strategies and interventions, with a goal of decreasing per capita alcohol consumption by 20% by 2030.

Among the various measures to reduce alcohol-related harm, increasing the price to reduce the affordability of alcohol has consistently shown to have the strongest impact.^9^^,^^10^ Minimum unit pricing (MUP) is a specific form of alcohol price control which targets cheaper and stronger alcohol products by setting a minimum price per unit of alcohol. It differs from excise duty, which can be absorbed by manufacturers and retailers. Scotland became a pioneer by introducing MUP in 2018,^9^^,^^10^ setting a floor price of £0.50 per United Kingdom (UK) unit of alcohol (i.e., 8 g), which was increased to £0.65 per unit as of September 2024.^11^ The implementation of this policy faced many delays due to alcohol industry-led legal challenges. A 2023 controlled interrupted time-series analysis compared alcohol-specific deaths and hospitalisations in Scotland after the introduction of MUP with those in England, which has no such policy. In the overall population, MUP was associated with a significant reduction in alcohol-attributable deaths by 13.4% and with a 4.1% decrease in hospitalisations.^12^ Moreover, among individuals with ALD, the study showed reductions in ALD-related deaths and hospitalisations by 11.7% and 9.8%, respectively.^12^

A common narrative promoted by the alcohol industry is that higher prices lead to increased consumption of illegal or unrecorded alcohol. However, recent evidence does not support this and, instead, suggests that alcohol pricing policies may reduce overall consumption, including unrecorded alcohol use, particularly when implemented with complementary.^13^ Moreover, although unrecorded alcohol accounts for approximately 25% of global alcohol consumption, this proportion varies widely geographically.^14^ In Europe, the prevalence of unrecorded alcohol use among drinkers ranges from around 2% to >25% depending on the country. These differences reflect variation in regulatory systems, informal production traditions, and cross-border markets. Consequently, any substitution effect following price increases is highly context-dependent and should be interpreted within specific national environments.^14^ Finally, despite concerns that MUP leads to harmful displacement of spending, increased crime or other substance misuse, these concerns have largely proven unfounded.^15^

MUP may have a limited impact on a subset of individuals who use alcohol and seem resistant to price-based interventions.^16^ Consequently, complementary policies are required. The effectiveness of alcohol policies is unlikely to be uniform across drinking profiles: population-level pricing and availability measures act broadly but may yield particularly large benefits among heavier drinkers, whereas screening, brief intervention, psychosocial and pharmacological treatments are more directly targeted to people with risky alcohol use and AUD.^17^ Large-scale implementation of SBIRT (Screening, Brief Intervention, and Referral to Treatment) in primary care can substantially reduce population-level alcohol consumption and related harms, but the effectiveness of this approach depends on adequate training of practitioners to achieve wide screening coverage and overcome implementation barriers.^18^ Simulation modelling in Germany suggested that increasing screening coverage to 50% of primary care patients could reduce national alcohol consumption by 11% over ten years, though coverage below 10% yields a minimal impact.^19^ The Service Capacity Index for Substance Use Disorders (SCI-SUD),^20^ which evaluates national treatment capacity for AUD, indicates that Europe has the highest mean SCI-SUD at 49%, with large cross-country variation.^1^ Despite this, access to AUD treatment remains low, with estimates placing it at 17.6% between 2013 and 2014, thereby undermining the effectiveness of interventions designed to increase engagement with alcohol-reduction programmes.^21^

From a public health perspective, the development of national strategies to reduce alcohol consumption and its consequences requires political and policymaker commitment, which is one of the most effective ways of improving population health.^22^ Notably, Lithuania's comprehensive alcohol control reforms, implemented between 2016 and 2018, which include excise tax increases, reduced sales hours, and a total ban on alcohol advertising, led to one of the steepest declines in per-capita alcohol consumption and alcohol-related mortality ever recorded in the Baltic region.^23^ Likewise, higher excise duties in Finland, advertising restrictions under France's Loi Évin, and retail availability controls in Nordic countries have shown measurable reductions in alcohol use and related harms. Although MUP has been the most intensively evaluated measure, these national examples collectively illustrate that strong, multisectoral policy packages may be preferable to yield significant public-health benefits (Table 1).

**Table 1:** Main evidence-based alcohol control measures endorsed by the World Health Organisation as “best buys” and their documented impact on alcohol-related harm, including liver disease mortality, in Europe.

Policy measure	Description	Evidence of effectiveness	Example(s)
Taxation/excise duty^23^	Increases retail price, reducing affordability	↓ consumption, ↓ liver and cardiovascular mortality	Finland, Lithuania
Minimum unit pricing^9^^,^^12^	Sets a minimum price per g or unit of alcohol	↓ alcohol-specific deaths (by −13.4%), ↓ alcohol-related liver disease deaths (by −11.7%)	Scotland (2018), Ireland (2023)
Advertising restrictions^24^	Limits exposure to alcohol marketing and sponsorship	↓ initiation among youth, ↓ overall consumption	France (Loi Évin), Lithuania
Availability restrictions	Regulates outlet density and sale hours	↓ per capita use and injuries	Nordic countries, Lithuania
Drink-driving enforcement	Low blood alcohol concentration limits, random breath testing	↓ road injuries and fatalities	European Union-wide initiatives

Although taxes can help offset the costs of alcohol-related health consequences, the financial burden of alcohol misuse clearly outweighs the income generated through taxes. For example, in Canada, the net deficit in 2020 was CAD $6.4 billion, with social costs of $19.7 billion compared with $13.3 billion in government revenue from alcohol sales.^25^ In the US, alcohol taxes cover only about 10% of the total economic cost of alcohol use, with the median total alcohol tax per drink ($0.21) accounting for just 10.3% of the median total economic cost per drink.^26^^,^^27^ Similarly, in the UK, annual costs from alcohol misuse are estimated at over £21 billion, far surpassing tax revenues.^6^

Beyond healthcare and fiscal policies, family and community-based initiatives are critical to reducing alcohol-related harm, particularly among adolescents. A well-documented example is the Icelandic Prevention Model, launched in the late 1990s.^28^ This nationwide strategy strengthened parental engagement, parental engagement, school–family collaboration, and investment in structured leisure activities. Over two decades, rates of adolescent alcohol intoxication declined markedly,^29^ demonstrating the potential of long-term, community-driven approaches to complement regulatory and healthcare-based strategies addressing social determinants of alcohol use.

### Barriers to the implementation of alcohol policies

The alcohol industry has strategically positioned itself as a stakeholder in policy debates and apparent but self-serving “public education” programmes, framed as “corporate social responsibility”.^30^^,^^31^ An example of this is the approach to marketing and advertising alcohol, which has been shown to increase alcohol consumption.^32^ Industry actors deploy tactics to delay, dilute, or derail evidence-based alcohol control measures.^33^ Through intense lobbying at national and European Union (EU) levels, the industry has opposed proposed public health regulations, including by challenging marketing restrictions and MUP legislation in courts and legislative forums. These tactics mirror those of the tobacco industry, creating doubt about evidence and emphasising personal responsibility over regulatory action.^33^ As a result, alcohol-related public health policies may have been weakened or abandoned, or inconsistently implemented.

A recent example of the alcohol industry's harmful influence on policy occurred in Ireland in 2025. In response to unsustainable levels of alcohol consumption and related harms, Ireland enacted the Public Health (Alcohol) Act in 2018. The Act introduced six evidence-based measures to reduce alcohol consumption and harms. Alcohol labelling, including specific warnings regarding alcohol causing liver disease and cancer and its consumption during pregnancy, was a key aspect of the Act, which passed through a series of legislative steps and was due to be implemented in May 2026. However, following intense lobbying, including the threats related to external tariffs on Ireland, the measure was deferred by the Irish Government deferred implementation for at least two years. This demonstrates nefarious interference of the alcohol industry and is an existential challenge to public health leaders, healthcare providers (HCPs), and the public to react to protect lives.

Minimum legal age laws for alcohol purchase and/or consumption are also established measures to reduce alcohol use and alcohol-related harms among adolescents and young adults.^34^ Across Europe, minimum legal age laws for alcohol purchase are universal but heterogeneous, typically set at 18 years, but with variation by country, beverage type, and drinking context, and with some countries adopting stricter thresholds such as 20 years.^35^ Moreover, although regulations on alcohol advertising are relevant, non-alcoholic beverage advertising might be a hidden form of alcohol advertising that is not subject to restrictions and has a measurable impact on attitudes toward alcohol, especially among young people. A randomised controlled trial in 2024 of 1638 UK regular alcohol consumers found that exposure to brand-matched alcohol-free and low-alcohol drinks increased brand (over product) recall.^36^

Another important factor is the lack of state- and EU-level apparatus that are accountable for alcohol consumption and its harms, and responsible for reducing them. For example, the European Alcohol Policy Alliance (Eurocare), a group of non-governmental organisations that advocates for alcohol-related policies, announced the closure of its office due to funding difficulties, underscoring how EU-level institutional support for alcohol policy and prevention is limited and insufficient.^37^ Considering the enormous societal toll of alcohol, a coordinated, unified state response of appropriate scale and influence is urgently required.

Cultural context substantially shapes the acceptability of alcohol policies. In particular, social, economic, demographic, cultural, and political factors are major drivers of drinking trends, and a “one-size-fits-all” approach to alcohol policy is unlikely to be optimal.^38^ Similarly, people are more likely to reject alcohol policies that do not align with the cultural determinants of drinking.^39^ Evidence specifically linking resistance to alcohol policies with public mistrust of government remains limited; instead, concerns appear to focus more on feasibility and potential unintended consequences than on distrust per se.^40^ At the same time, greater awareness of alcohol's health effects, including its association with cancer risk, has been linked to increased support for population-level alcohol policies.^41^

ALD disproportionately affects socioeconomically disadvantaged populations, a pattern described as the ‘alcohol harm paradox’.^42^ A meta-analysis confirmed a dose–response relationship between socioeconomic deprivation and alcohol-attributable mortality,^43^ yet differences in alcohol consumption explain only up to 27% of these inequalities.^44^ These inequalities are driven by structural factors including income insecurity, precarious employment, chronic stress, and unequal access to prevention and care. Framing ALD solely as an individual behavioural issue risks obscuring its strong social gradient and the need for equity-oriented policies. Within Europe, the feasibility, enforcement, and likely impact of alcohol policies often differ across lower-, middle-, and high-income settings, reflecting variation in regulatory infrastructure, health-system capacity, baseline preparedness, and exposure to industry interference; therefore, implementation strategies should be locally adapted.

### Alcohol-related preparedness and education deficits

**Fig. 2:**
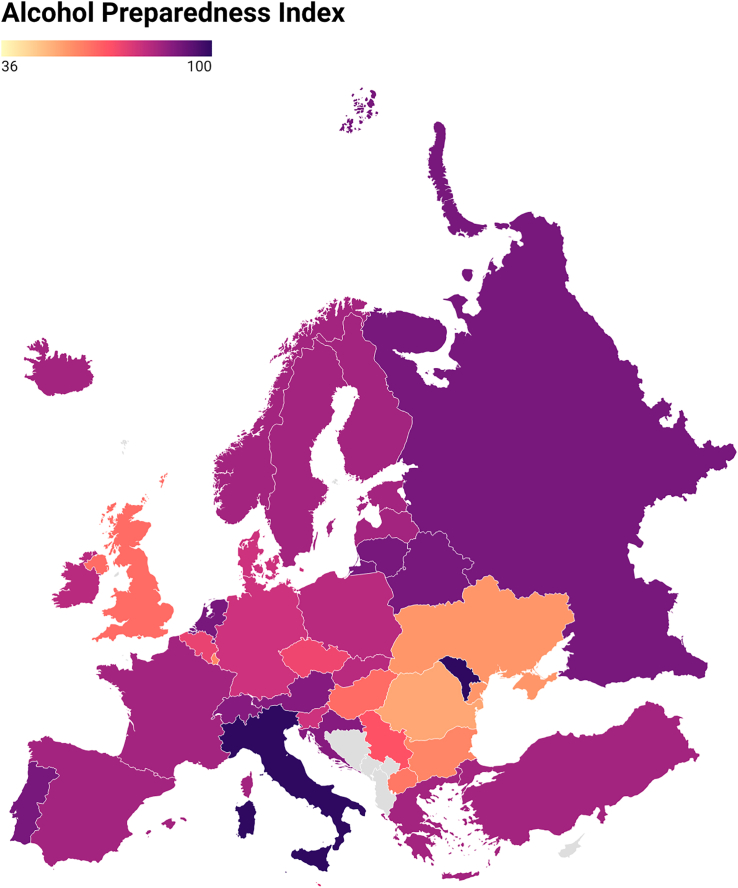
Heatmap of the Alcohol Preparedness Index obtained for each European country in 2019. The preparedness index ranges from 0 to 100, with 0 representing the lowest preparedness and 100 the highest.

In the face of Europe's high ALD burden, its readiness to implement an effective alcohol control can be assessed via tools like the alcohol preparedness index (API).^45^ Although the region's median API score increased between 2010 and 2019, updated analyses show uneven progress. Less than 25% of countries have a strong national alcohol action plan and 40% have not drafted any such plan, demonstrating considerable gaps in preparedness and implementation (Fig. 2). Western and northern European nations improved alcohol-related preparedness over the 2010s, improving alignment with WHO best buy interventions, whereas some eastern and southern countries lagged behind. Importantly, larger-population countries (e.g., Germany, Spain, and the UK) show smaller preparedness improvements, suggesting greater implementation challenges. Critically, nations with more robust alcohol control policies demonstrated substantially lower rates of ALD, hepatocellular carcinoma, and cardiovascular mortality attributable to alcohol.^45^ Given the proven link between alcohol policy implementation and reduced alcohol harms that there is an urgent need to strengthen implementation.

In addition to policy implementation, awareness and education around alcohol harms are a crucial aspect of preparedness. Yet, an online survey conducted among adults who consume alcohol across 14 European countries found that only about half of respondents were aware of the link between alcohol and cancer.^46^ This lack of awareness extends to ALD, as many individuals still perceive cirrhosis as a rare outcome or believe that only people living with an AUD get liver disease, underestimating the risk from even moderate chronic alcohol intake. Insufficient medical education (including destigmatisation of alcohol use) is problematic as HCPs across Europe receive variable training in substance use medicine and ALD management. Although public awareness of the link between alcohol use and liver disease is high, with 96.3% of respondents acknowledging the link between alcohol consumption and liver disease in an Irish survey, a high percentage of respondents still perceived regular or high-volume alcohol use as socially acceptable, highlighting the persistent gap between knowledge and behaviour.^47^

## Stigma toward people living with high-risk alcohol use and ALD in Europe

Stigma surrounding AUD and ALD is a pervasive issue, rooted in stereotypes and often exacerbated by media portrayals and low health literacy.^48^^,^^49^ Among individuals living with alcohol intake patterns that place them at risk of liver disease, stigma operates on multiple levels: within themselves, societally via relationships (e.g., with family or at work), within healthcare settings, and politically. Such self-, public, and structural stigma has been documented in ALD, contributing to discrimination, delayed care, and suboptimal health outcomes.

Stigma is frequently experienced by people living with liver disease, with 73% of1134 such individuals reporting having experienced it in a UK survey.^50^ This leads to care avoidance, contributing to the burden of ALD. In fact, individuals living with severe self-stigma present with a 6-fold risk of care avoidance.^51^ Moreover, stigma may be compounded by mental health comorbidities, which are highly prevalent among people living with ALD^52^ and are themselves stigmatised.^53^ Evidence from mental health research suggests that stigma is reduced most effectively through interventions that combine education with direct contact with people with lived experience.^53^ Advocacy, patient empowerment, and strong social support networks also appear to be beneficial.

## Population level interventions to reduce alcohol-related harm in Europe

Efforts to reduce ALD in Europe require multi-level strategies integrating robust alcohol control policies, early identification of risky alcohol intake, anti-stigma interventions to facilitate care commitment, and effective treatment for AUD.^33^^,^^54^ Even brief counselling sessions enable meaningful reductions in alcohol intake which, when scaled, have the potential to translate into significant public health benefits.^55^ Yet, several European countries underperform in systematic alcohol screening, leaving major gaps in preparedness. For individuals living with ALD, access to evidence-based therapies, including pharmacological agents such as naltrexone, baclofen, and acamprosate, combined with psychosocial support, is life-saving.^56^ However, treatment coverage in Europe remains <10%, constrained by stigma and care service fragmentation. A comprehensive approach, combining strong alcohol pricing and marketing policies, widespread SBIRT, and expanded access to AUD treatment, would yield measurable population-level outcomes including lower per-capita alcohol use, fewer hospitalisations for alcohol-related hepatitis and cirrhosis, and reduced liver-related mortality. If even a modest replication of the Scottish post-MUP mortality reduction was achieved, thousands of lives could be saved annually.

## Screening programmes, tools for diagnosis, and pathways for referral

**Fig. 3:**
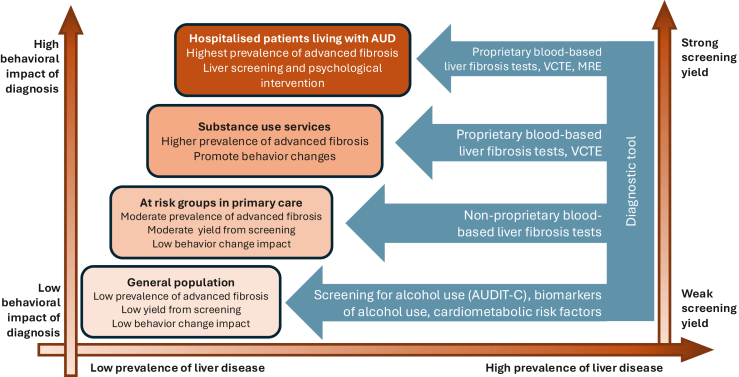
Conceptual framework for screening of alcohol-related liver disease and metabolic dysfunction-associated alcohol-related liver disease (MetALD) across prioritised populations. As the priority population narrows from the general population to the at-risk population in primary care, substance use units, and hospitalised inpatients living with an alcohol use disorder, the prevalence of advanced liver disease increases, as does the screening yield of strategies for the detection of liver disease and the behavioural impact of a diagnosis. Abbreviations: AUDIT, Alcohol Use Disorder Test; AUDIT-C, Alcohol Use Disorder Test: Consumption; VCTE, vibration-controlled transient elastography; MRE, magnetic resonance elastography.

Data from one of the largest European liver disease screening programmes, which included >30,000 individuals across nine countries, showed that 4.6% and <3% of people had significant and advanced fibrosis, respectively.^57^ The likelihood of advanced fibrosis was substantially higher in individuals living with metabolic risk factors and/or high-risk alcohol use but remained relatively low otherwise. These findings support guideline recommendations for targeted case-finding among high-risk groups rather than universal screening, especially those living with an AUD and/or metabolic dysfunction, where structured diagnostic pathways have shown to be cost-effective (Fig. 3).^58^

### Screening tools for AUD and ALD

Quantification of alcohol intake is commonly under-reported by patients and thus underestimated by clinicians.^56^ Thus, alcohol intake, in grams per week, and drinking patterns (e.g., daily versus bingeing) should be assessed in all relevant healthcare encounters and documented in electronic health records. Screening for high-risk alcohol intake should begin with a brief questionnaire such as the Alcohol Use Disorders Identification Test: Consumption (AUDIT-C), where a positive AUDIT-C result should prompt the undertaking of the full AUDIT.^56^ In a large trial (ODHIN) that included more than 36,000 participants in five countries, the AUDIT-C showed high accuracy, but many individuals who screened positive for risky consumption initially did not receive an intervention.^59^^,^^60^ However, the EASY trial found no reduction in alcohol intake after an ultra-brief (i.e., <1 min) physician intervention, suggesting that such minimal contact is insufficient to foster behavioural change.^61^ Identification of alcohol use can be complemented by objective biomarkers like urine-based (ethyl glucuronide -EtG- and ethyl sulphate -EtS-) or blood-based (phosphatidylethanol -PEth-) to mitigate under-detection.^62^^,^^63^ PEth reflects alcohol use over approximately four weeks and accurately identifies a sustained intake,^64^ making it a promising biomarker for the identification of metabolic dysfunction-associated alcohol-related liver disease (MetALD) and ALD. A recently developed scalable biomarker panel has also demonstrated good performance in predicting these conditions in individuals with MetALD and ALD, and may serve as an alternative in settings where alcohol biomarkers are not available.^65^

Because alcohol is a leading cause of liver fibrosis, fibrosis assessment should be undertaken in people living with high alcohol use per clinical guidelines.^66^ Screening for alcohol use is also recommended in individuals living with type 2 diabetes, obesity, or suspected metabolic dysfunction-associated steatotic liver disease (MASLD), given the strong interaction between cardiometabolic risk factors and alcohol in liver morbidity. Notably, alcohol intake is often under-reported among patients presumed to be living with MASLD, with significant consumption in ∼30% of patients.^62^ Over time and with changes in consumption, up to 38% undergo a shift in intake category, as defined within the steatotic liver disease spectrum.^67^ Thus, repeated assessments of alcohol consumption are central for accurate diagnosing and management. Fig. 3 shows a framework for populations that should be prioritised for ALD and MetALD screening, the tools to be used, and the effects of screening at different levels.

### Potential beneficial effects and barriers for the implementation of ALD and MetALD screening programmes

Screening programmes for ALD and MetALD show benefits, particularly among individuals living with high-risk alcohol use or metabolic dysfunction. These may enable earlier fibrosis detection, facilitate behavioural change, and reduce health complications and costs.^68^^,^^69^ Such screening can also promote an alcohol intake reduction^70^; in a Barcelona substance use unit study, abstinence six months post screening was higher among screened individuals than controls (45% versus 29%).^71^ A Danish programme also found sustained decreased alcohol intake among those who tested positive for fibrosis.^72^

Implementation of these screening programmes faces structural barriers, including variability in healthcare organisation, reimbursement, and national priorities.^73^ In well-resourced systems, fragmented care provision may limit screening coordination, while implementation is hindered in lower-means settings due to resource constraints and competing priorities. Stigma, low health literacy, and limited public awareness of liver disease as a preventable condition further reduce patient engagement.^68^^,^^74^ Integrating screening into routine health checks and developing coordinated, well-funded pathways are needed to improve its uptake and effectiveness.

### Integrated and multidisciplinary models of care for ALD and MetALD

**Fig. 4:**
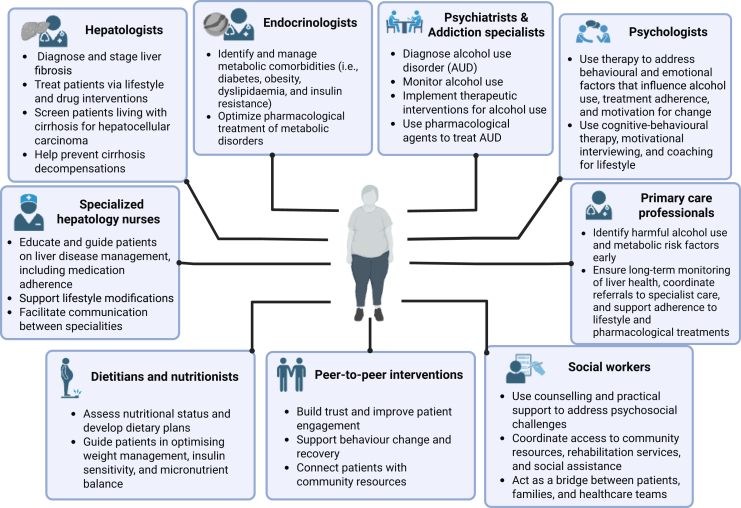
Multidisciplinary model of care for patients living with alcohol-related liver disease and metabolic dysfunction-associated alcohol-related liver disease. The figure illustrates the key healthcare professionals involved in an integrated, patient-centred model of care, including primary care physicians, hepatologists, endocrinologists, addiction specialists, psychologists, dietitians, specialised nurses and primary care physicians, endocrinologists, psychologists, specialised hepatology nurses, dietitians and social workers. Abbreviations: ALD, alcohol-related liver disease; MetALD, metabolic dysfunction-associated alcohol-related liver disease.

AUD and high-risk alcohol use are highly prevalent among individuals living with MetALD and ALD. In a population-based study, nearly 80% of participants living with these conditions had intermediate or high AUDIT-C scores and corresponding PEth concentrations, indicating ongoing alcohol intake.^75^ These findings highlight that management of AUD must be systematically integrated within liver disease care, as treating one without the other rarely achieves sustained benefits (Fig. 4).

Emerging care models range from coordinated referral networks to fully integrated clinics combining hepatology and substance use services.^76^ Pilot initiatives, such as the multidisciplinary ALD clinic described by Mellinger et al., demonstrate that shared care among hepatologists, psychiatrists, psychologists, nurses, and social workers can reduce healthcare visits and improve patient satisfaction.^77^ Although most data come from small or non-randomised studies, these programmes suggest that integrated pathways can improve alcohol abstinence, reduce healthcare readmissions, and strengthen follow-up.^76^

The elevated prevalence of cardiometabolic risk factors in people living with ALD adds complexity, reinforcing the need for multidisciplinary teams including hepatologists, substance use specialists, endocrinologists, and specialist nurses. Despite the resources required, such integrated models of care have shown benefits for ALD and MASLD and represent a promising framework for comprehensive liver care (Fig. 4).^78^ Integration of peer support workers into multidisciplinary clinics may further enhance patient engagement, trust, and treatment adherence, particularly among individuals living with mental disorders and AUD. Evidence from substance use clinics and HIV and hepatitis C programmes shows that peer-led interventions improve linkage to care, care retention, and treatment outcomes,^79^^,^^80^ suggesting that similar approaches could strengthen the effectiveness and reach of integrated ALD and MetALD clinics.

### Digital health tools and innovative solutions for multidisciplinary clinics for an AUD, ALD, and MetALD

Digital health tools, particularly smartphone applications, offer a promising approach to monitor risk factors and deliver personalised interventions, while requiring minimal patient effort and enabling real-time data collection and biofeedback. The combination of multidisciplinary clinical care with digital monitoring tools could be particularly advantageous for MetALD, allowing continuous monitoring of metabolic and behavioural risk factors and enabling early intervention. Harnessing interprofessional collaboration and technological innovation will be essential in delivering patient-centred care for populations living with ALD or MetALD.

## Future directions, recommendations, and research priorities

### Social education and programmes for young populations

Children and adolescents have been failed by societies in most European countries, which have fallen short in protecting them from alcohol-related harms, including foetal alcohol spectrum disorders and parental alcohol misuse, adverse childhood experiences, and early exposure to alcohol. Data from the Health Behaviour in School-aged Children study indicate that over 50% of 15-year-olds have consumed alcohol, and that 20% have risky alcohol use patterns, indicating a societal failure of protection.^81^ Preventing ALD requires a life-course approach beginning in childhood, when social norms and self–regulatory processes are still developing.^82^ Traditional awareness campaigns, largely based on information- or fear-based messaging, have produced modest behavioural effects and limited engagement.^83^

Innovative prevention models grounded in behavioural and digital science offer promising care alternatives. Behavioural games can train individuals in emotional regulation, the delay of gratification, and resistance to peer pressure, while maintaining high user engagement.^84^ A recent meta-analysis put into evidence that classroom behaviour-management strategies like the Good Behaviour Game (GBG), implemented in early school years can decrease addictive behaviours, underscoring the importance of early universal prevention approaches.^85^ Gamified interventions and interactive educational platforms, co-designed with adolescents, have shown preliminary efficacy in modifying attitudes toward alcohol and other substance misuse behaviours.^86^ Social media can also serve as a vector for peer-led health promotion and the diffusion of prosocial norms. However, social media exposure to alcohol-related content, including posts by peers and brands, may increase alcohol use and the normalisation of drinking behaviours, particularly among young people.

### Policy reform opportunities

Population-level measures are the most powerful interventions to reduce alcohol-related harms (Panel 1). Fiscal and regulatory interventions, particularly increased taxation and MUP, consistently decrease alcohol consumption and its related hospitalisations and mortality, including from liver disease.^87^ Complementary policies restricting advertising, sponsorship, and digital marketing targeting youth are essential to counter the normalisation of alcohol in contemporary culture.^88^ Labels highlighting such risks can be effective in discouraging risky alcohol use patterns.^89^

Panel 1 **Recommendations for action to address alcohol-related outcomes.**

1 Implement evidence-based alcohol control policies at scale.
  a European governments should fully adopt and implement the World Health Organisation alcohol-related “best buys”, including three alcohol-specific NCD “quick buys” with the potential to deliver measurable public health impact within 5 years: increased excise taxes on alcoholic beverages, restrictions on the physical availability of retailed alcohol, and restrictions on exposure to alcohol advertising. Complementary pricing policies, including minimum unit pricing, should also be considered.
  b Ensure index-linked pricing and reinvest alcohol revenue into public health initiatives.
2 Establish independent national and European Union-wide bodies that are accountable for alcohol harm.
  a A dedicated public health agency or programme, free from industry influence, should monitor alcohol consumption at a population level and its health and social costs, and develop and implement cross-sector policies, funded by an excise duty levy on the alcohol industry.
3 Enforce transparency and prohibit alcohol industry interference in health policy.
  a The alcohol industry should be excluded from policy development and health communication.
  b Binding lobbying transparency and conflict-of-interest frameworks, like those for tobacco, are essential to protect evidence-based policymaking.
4 Embed alcohol risk awareness and stigma reduction in education and the media.
  a Public information campaigns should highlight alcohol's causal link to liver disease, cancer, and other harms, while incorporating contact-based anti-stigma interventions to humanise alcohol use disorder (AUD), alcohol-related liver disease (ALD), and metabolic dysfunction-associated alcohol-related liver disease (MetALD).
5 Strengthen preparedness through training and health system capacity.
  a Healthcare training across Europe should integrate structured education in substance use medicine, early detection of liver disease, and stigma-free care provision, to contribute to preparedness for managing an AUD, ALD, and MetALD.
6 Integrate systematic alcohol use screening and related early interventions into primary care.
  a Primary care and community settings should routinely apply the Alcohol Use Disorders Identification Test (AUDIT) questionnaire and related brief interventions, complemented by fibrosis assessment using non-invasive biomarkers among individuals at high risk of ALD alcohol intake patterns and/or metabolic risk factors.
7 Develop and test the clinical effectiveness of multidisciplinary clinics for an AUD, ALD, and MetALD.
  a Health systems should promote integrated hepatology, substance use, and metabolic clinics, engaging hepatologists, substance use specialists, endocrinologists, psychologists, dietitians, nurses, and social workers to deliver coordinated care and improve health outcomes.
8 Leverage digital health tools to support health monitoring and behaviour change.
  a Mobile applications and telehealth platforms should be harnessed to track alcohol use and metabolic risk, deliver personalised feedback, support alcohol craving management, and enhance adherence to care.
9 Empower communities and people with lived experience.
  a Patient- and peer-led initiatives should be supported to combat stigma, enhance care engagement, and promote long-term alcohol use recovery and social reintegration.
10 Invest in research on implementation, equity, and long-term outcomes.
  a Future research should evaluate the real-world effectiveness and cost-effectiveness of alcohol policies, screening strategies, and multidisciplinary care models, including their impact on morbidity, survival, and years of working life lost due to ALD and MetALD.

Future policy research must move beyond theory to real-world evaluation, focussing on factors like implementation, equity, and cost-effectiveness across diverse contexts. Moreover, clearer timeframes for the implementation of alcohol-control policies are needed to guide governments in prioritising implementation and resource allocation. Sustained advocacy and clear communication are also paramount to keeping ALD and MetALD on the public health agenda.

### Current and future research priorities

Clinical research should prioritise early detection, focussing on non-invasive biomarkers and imaging modalities that are capable of identifying subclinical disease.^7^ There is a major unmet need for effective treatments in MetALD and ALD. Currently, four active Phase 2 trials are evaluating different drugs—each also being studied for MASLD—in patients living with MetALD and ALD (National Clinical Trial [NCT] identifiers: NCT06409130, NCT06613698, NCT07046819, and NCT07009860). Trials designed to include underrepresented groups—especially women and individuals living with metabolic comorbidities—are needed for the generalisability of findings and care equity.

More implementation research is needed to better assess conditions making classroom interventions effective on risky patterns of alcohol use in the long term.^85^ Digital and artificial intelligence-based tools may improve risk stratification, support personalised care, and enhance long-term disease monitoring and relapse prevention, bridging clinical care and self-management.^90^ Pharmacological research should continue exploring molecular pathways implicated in alcohol-related liver injury, ideally within multidisciplinary frameworks combining biomedical and psychosocial approaches. For example, glucagon-like peptide-1 receptor agonists and other incretin-based therapies, such as semaglutide and tirzepatide, which are commonly used to treat type 2 diabetes and obesity, may reduce alcohol use and possibly prevent AUD in some settings.^91^, ^92^, ^93^ In parallel, community-led interventions involving individuals with lived experience of alcohol use challenges can enhance engagement, reduce stigma, and promote social reintegration.^7^ Collaborative, international research remains essential for scalable and globally relevant innovations.

Future progress in reducing ALD will depend on synergistic action across educational, policy, and research domains. Embedding liver health, including preventive hepatology, within broader NCD strategies will ensure sustained visibility and integration into preventive care.^94^ Primary healthcare settings should routinely screen for hazardous alcohol intake using validated tools, coupled with structured referral pathways to psychosocial and substance use services.^7^ Policy reform must also address the social determinants of alcohol use—such as poverty, housing instability, and mental distress—through multisectoral collaboration.^7^

## Conclusions

Europe continues to face a substantial yet preventable burden of ALD and MetALD, driven by persistently high alcohol consumption, delayed diagnosis, weak health system preparedness, and inadequate implementation of proven alcohol control policies. The principal challenge is the failure to implement evidence-based policies routinely and consistently, in part due to the strong influence of the alcohol industry on policy and public discourse. A coordinated European response is essential to reduce the growing burden of ALD and MetALD and improve liver health and extrahepatic outcomes across the region.

## Contributors

EPM, LAD, JVL, PC, and PNB conceived the manuscript. All authors participated in drafting the first version of the article and reviewed and approved the final version.

## Editor note

The Lancet Group takes a neutral position with respect to territorial claims in published maps and institutional affiliations.

## Declaration of interests

EPM declares scientific advisory fees from GSK and Boehringer-Ingelheim, outside of this work. LAD declares speaking fee honoraria from Novo Nordisk, outside of this work. AK has served as speaker and/or consultant/on an advisory board for Novo Nordisk, Norgine, Boehringer Ingelheim, GSK, W4Cure, and Madrigal; he has received research support to his institution from Astra, Siemens, Nordic Bioscience, GSK, and Echosense; he is board member and co-founder of Evido; this is all outside of this work. JVL acknowledges grants to his institutions from AbbVie, Boehringer Ingelheim, Echosens, Gilead Sciences, Madrigal Pharmaceuticals, Moderna, MSD, Novo Nordisk, Pfizer, and Roche Diagnostics, consulting fees from Echosens, GSK, Madrigal Pharmaceuticals, Novo Nordisk, Pfizer, Sonic Incytes, and Takeda and honoraria for lectures from Echosens, GSK, Janssen, MSD, Novo Nordisk, and Pfizer, outside of this work. RB acknowledges consulting fees from Boehringer Ingelheim, Novo Nordisk, and GSK outside of this work. PNB acknowledges consulting fees from Boehringer Ingelheim, Madrigal Pharmaceuticals, Novo Nordisk and Resolution Therapeutics, as well as educational honoraria from Takeda and Novo Nordisk outside of this work.
